# *Notes from the Field:* School-Based and Laboratory-Based Reporting of Positive COVID-19 Test Results Among School-Aged Children — New York, September 11, 2021–April 29, 2022

**DOI:** 10.15585/mmwr.mm7132a2

**Published:** 2022-08-12

**Authors:** Eric J. Shircliff, Eli S. Rosenberg, Lauren M. Collens, Dina Hoefer, Emily Lutterloh, Benjamin J. Silk, Amber K. Winn, Travis T. O’Donnell

**Affiliations:** ^1^New York State Department of Health; ^2^School of Public Health, University at Albany, State University of New York, Rensselaer, New York; ^3^Division of Viral Diseases, National Center for Immunization and Respiratory Diseases, CDC.

By April 29, 2022, a total of 702,686 COVID-19 cases were reported among children and adolescents aged 5–17 years in the state of New York.* Pediatric COVID-19 cases and hospitalizations increased during the 2021–22 school year, driven by transmission of the Omicron variant^†^ (*1*). In late 2021, during the surge in Omicron BA.1 variant cases, state^§^ and federal^¶^ authorities expanded access to self-administered, at-home rapid antigen tests, which can increase a person’s knowledge of their COVID-19 status and guide risk-reduction behaviors. New York government agencies sent millions of these tests to schools for distribution to teachers, students, and staff members. Because results of self-administered, at-home tests are not captured by electronic laboratory reporting (in contrast to health care provider–administered tests at a physician’s office or laboratory that are reported through electronic health records or other means), expanded use of these tests might affect interpretation of trends in reported COVID-19 cases; however, this has yet to be assessed** (*2*). Furthermore, understanding changes in testing behavior before and after the Omicron variant surge might help public health officials better use available COVID-19 data to guide future policy.

COVID-19 case data from two independently operating New York State Department of Health systems were compared before and after expansion of at-home testing: 1) laboratory-reported data^††^ for children and adolescents aged 5–17 years and 2) a kindergarten through grade 12 (K–12) school-based system^§§^ for reporting positive results from all testing sources^¶¶^ (*3*). Laboratory-reported data include results of school-administered tests (which are required to be reported) but exclude results from self-administered, at-home tests. School-reported data include positive results reported to the state from any test source, including those from clinical settings, school-based testing programs, and self-administered, at-home tests. Case totals for both data sets*** and the ratio of school-reported to laboratory-reported cases were calculated weekly during September 11, 2021–April 29, 2022, and compared. This activity was reviewed by CDC and was conducted consistent with applicable federal law and CDC policy.^†††^

During the September 11–17, 2021, school week, among 6,928 New York schools, 5,201 (75.1%) reported to the school-based system; by the April 23–29, 2022, school week, 5,274 (76.1%) schools reported (weekly median = 80.7%; IQR = 76.1%–81.7%). During the entire analysis period, 477,538 student cases were reported to the K–12 school-based system, and 464,421 cases in children and adolescents aged 5–17 years were reported by laboratories^§§§^; the overall ratio of school-reported to laboratory-reported cases was 1.03. During September 11–December 31, 2021, the ratio of school-reported to laboratory-reported cases was stable and near 1.0 (median = 0.82; IQR = 0.73–0.85) (Figure). From the January 1–7 to the April 29, 2022, school week, during and following state and federal expansion of at-home testing, the ratio of school-reported to laboratory-reported cases increased 167%, from 1.36 to 3.64 (median = 1.58; IQR = 1.36–2.13).

**FIGURE:**
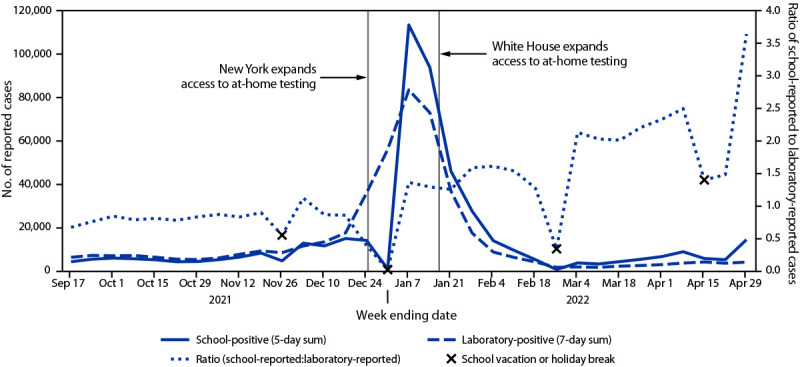
School-reported* and laboratory-reported^†^ COVID-19 cases — New York, September 11, 2021–April 29, 2022 * School-reported data include positive results from any test source, reported through the New York state COVID-19 report card system for children in kindergarten through grade 12. ^†^ Laboratory-reported data include positive results of SARS-CoV-2 reverse transcription–polymerase chain reaction and antigen tests conducted at laboratories or physician offices, reported through electronic health records or other means.

These findings are subject to at least three limitations. First, because school-reported data include some students aged <5 years or >17 years, and not all children and adolescents aged 5–17 years attend schools that reported cases, school-reported and laboratory-reported case data were not directly comparable. Second, these results might reflect both underreporting of infection and increased detection because of at-home test use. Finally, results from school-aged children and adolescents are not representative of those from the general population.

The changing relationship between school-reported and laboratory-reported data, during a period of stable school reporting, suggests a decline in the capture of positive laboratory test result data for children and adolescents aged 5–17 years following the expansion of at-home testing. Throughout the pandemic, public health programs have relied on laboratory-reported data to guide risk communication; underestimation of cases based on these data could affect interpretations of epidemic trends and metrics derived from them, including community COVID-19 incidence. This analysis suggests that methods of capturing data on results from self-administered, at-home tests can augment laboratory-reported data to provide a more complete picture of positive COVID-19 test results within communities. Jurisdictions that prioritize both at-home COVID-19 testing and comprehensive epidemiologic monitoring of the COVID-19 pandemic might consider implementing reporting systems that operate alongside electronic laboratory reporting. As the pandemic has evolved, however, the level of vaccine- and infection-derived immunity has increased in the population; thus, prioritization of reducing medically significant illness and minimizing strain on the health care system has increased.^¶¶¶^ Health officials and the public should consider current information about COVID-19 cases and hospitalizations in the community, as well as the potential for strain on the local health system, when making decisions about community prevention strategies and individual behaviors.****

## Footnotes

*
https://coronavirus.health.ny.gov/covid-19-data-new-york

†
https://coronavirus.health.ny.gov/pediatric-covid-19-update-january-21-2022

§
https://www.governor.ny.gov/news/video-audio-photos-rush-transcript-governor-hochul-announces-comprehensive-winter-surge-plan

¶
https://www.whitehouse.gov/briefing-room/statements-releases/2022/01/14/fact-sheet-the-biden-administration-to-begin-distributing-at-home-rapid-covid-19-tests-to-americans-for-free/

**
https://www.cdc.gov/coronavirus/2019-ncov/testing/self-testing.html

†† Laboratories in New York state report results from both reverse transcription–polymerase chain reaction and antigen tests.

§§ Since September 2020, all K–12 schools have been required to submit data on the number of students, teachers, and staff members who have reported receiving positive COVID-19 test results by 5:00 p.m. each day (excluding weekends, vacation breaks, and unexpected closures). https://schoolcovidreportcard.health.ny.gov/

¶¶ Schools report any notification of positive test results to the New York State Department of Health from a variety of sources, including school-based testing programs, results from community-based diagnostic and at-home testing reporting by families and providers, and notifications from a local health department as part of contact tracing efforts.

*** The number of school-reported cases is typically higher on Mondays because of the cumulative caseload from the preceding weekend. Therefore, 5-day weekly sums for schools were compared with 7-day weekly sums for laboratories, (e.g., Monday, September 13, 2021–Friday September 17, 2021, for school-reported data and Saturday, September 11, 2021–Friday, September 17, 2021, for laboratory-reported data). Both data sets are statewide and include New York City.

††† 45 C.F.R. part 46.102(l)(2), 21 C.F.R. part 56; 42 U.S.C. Sect. 241(d); 5 U.S.C. Sect. 552a; 44 U.S.C. Sect. 3501 et seq.

§§§ Laboratories in New York state are required to submit COVID-19 test results only if they receive specimens for testing. In 2021, the compliance rate for all laboratory facilities was 95.6%.

¶¶¶
https://www.cdc.gov/coronavirus/2019-ncov/science/science-briefs/indicators-monitoring-community-levels.html

****
https://www.cdc.gov/coronavirus/2019-ncov/science/community-levels.html

## References

[1] Shi DS, Whitaker M, Marks KJ, ; COVID-NET Surveillance Team. Hospitalizations of children aged 5–11 years with laboratory-confirmed COVID-19—COVID-NET, 14 states, March 2020–February 2022. MMWR Morb Mortal Wkly Rep 2022;71:574–81. 10.15585/mmwr.mm7116e1 35446827 PMC9042359

[2] Rader B, Gertz A, Iuliano AD, Use of at-home COVID-19 tests—United States, August 23, 2021–March 12, 2022. MMWR Morb Mortal Wkly Rep 2022;71:489–94. 10.15585/mmwr.mm7113e1 35358168 PMC8979595

[3] Rosenberg ES, Dufort EM, Blog DS, ; New York State Coronavirus 2019 Response Team. COVID-19 testing, epidemic features, hospital outcomes, and household prevalence, New York State—March 2020. Clin Infect Dis 2020;71:1953–9. 10.1093/cid/ciaa549 32382743 PMC7239264

